# Hip Abductor Strength Predicts Injurious Falls and Mediates the Balance Confidence–Falls Relationship: A Competing Risk Model

**DOI:** 10.1002/jcsm.70280

**Published:** 2026-04-01

**Authors:** Tewodros Yosef, Julie A. Pasco, Monica C. Tembo, Kara L. Holloway‐Kew

**Affiliations:** ^1^ School of Medicine, IMPACT, The Institute for Mental and Physical Health and Clinical Translation Deakin University Geelong Victoria Australia; ^2^ School of Public Health, College of Medicine and Health Sciences Mizan‐Tepi University Mizan Teferi Ethiopia; ^3^ Department of Medicine—Western Health The University of Melbourne St Albans Victoria Australia; ^4^ Department of Epidemiology and Preventive Medicine Monash University Melbourne Victoria Australia

**Keywords:** aged, balance confidence, hip abductor strength, injurious falls, sarcopenia, specific force

## Abstract

**Background:**

Falls are a major public health issue, largely driven by age‐related declines in hip and lower limb muscle strength. Hip muscle strength plays a critical role in postural stability and falls prevention. Lower balance confidence increases fall risk by restricting activity participation, which may contribute to muscle weakness over time. This study examined the association between hip abductor and flexor strength and the incidence of injurious falls in older adults and investigated whether hip abductor and flexor strength mediate the relationship between balance confidence and incident injurious falls.

**Methods:**

Participants (*n* = 952; aged ≥ 65 years) were drawn from the Geelong Osteoporosis Study (GOS). The outcome was the time to first emergency department presentation for an incident injurious fall. Hip abductor and flexor strength were assessed using a handheld dynamometer to measure hip abduction and flexion force, with strength values adjusted for leg lean mass measured by dual‐energy X‐ray absorptiometry. Balance confidence was assessed using the 14‐item Modified Falls Efficacy Scale (MFES). Associations between hip muscle strength and incident injurious falls were evaluated using a competing risk regression model, which accounted for death as a competing event. The results are expressed as adjusted sub‐distribution hazard ratios (aSHR) and 95% confidence intervals. Mediation analysis was conducted to assess whether hip abductor and flexor strength mediated the relationship between balance confidence and the incidence of injurious falls.

**Results:**

Among the 952 participants, 38% were women (mean age 76.1 ± 7.3 years), and 62% were men (mean age 76.9 ± 7.0 years). The median follow‐up time was 11.5 years (IQR 5.9–19.0). During follow‐up, 219 participants (23.0%) experienced at least one injurious fall, corresponding to an incidence rate of 19.3 per 1000 person‐years (95% CI: 16.9–22.0). Greater hip abductor strength was associated with a lower risk of incident injurious falls (aSHR = 0.835, 95% CI: 0.724–0.963; *p* = 0.013), with each 1‐N/kg increase in hip abductor strength reducing the sub‐distribution hazard by 16.5%. Hip flexor strength was not significantly associated with incident injurious falls. Hip abductor strength accounted for 23.7% of the association between balance confidence and incident injurious falls.

**Conclusions:**

Greater hip abductor strength is protective against incident injurious falls in older adults and partially mediates the relationship between balance confidence and injurious falls. Fall prevention strategies should integrate hip abductor strengthening with interventions targeting cognitive and psychological factors, such as improving balance confidence.

## 1 Introduction

Falls are a major public health concern [1, 2], often resulting in serious injury or death [3]. Each year, nearly one in three adults aged ≥ 65 years report a fall, increasing to one in two among those aged ≥ 80 years [4]. Falls rank as the fifth leading cause of death in older adults and are largely driven by modifiable risk factors [5], particularly skeletal muscle strength, which is central to balance, mobility and daily activities [6, 7]. Ageing leads to musculoskeletal decline through changes in body composition, reduced muscle strength, decreased flexibility and impaired balance [8, 9].

Muscle strength declines by 3.6%–5.0% annually after age 65 [10], at a rate greater than the loss of muscle mass [11]. This decline contributes to gait impairment, sarcopenia and reduced independence [11, 12]. Sarcopenia, marked by a loss of muscle size and strength due to fibre shrinkage and loss [12, 13], is a recognized determinant of falls. Sarcopenia, defined by international consensus groups [14, 15, 16], though estimates vary depending on the criteria applied [17]. While both muscle strength and mass decline with age, strength has consistently proven to be the stronger predictor of falls [18, 19, 20, 21, 22, 23].

Falls in older adults are strongly associated with poor balance and muscle weakness [24], with hip muscle strength (HMS) playing a very critical role in stability [25]. Evidence indicates that stronger HMS reduces fall risk, yet it is often overlooked in clinical assessments [25]. Low balance confidence is another powerful predictor that not only elevates fall risk but also may accelerate physical decline by limiting activity, reducing muscle strength and ultimately increasing vulnerability to falls [26].

Both lower limb muscle strength and low balance confidence have been identified as important predictors of falls. However, little is known about the specific contribution of hip abductor and flexor strength, or whether these muscle groups mediate the relationship between balance confidence and the incidence of injurious falls. By examining this interplay, the present study addresses a key gap in the literature. It provides evidence on how psychological and physical factors jointly shape fall risk, with important implications for integrated prevention strategies that strengthen both mind and muscle.

## 2 Methods

### 2.1 Participants and Data Source

Participants (*n* = 952, age ≥ 65 years) were from the Geelong Osteoporosis Study (GOS) [27]. The GOS is a longitudinal study that recruited participants from the Barwon Statistical Division in southeastern Australia. Participants were randomly selected from the Australian electoral roll to ensure an even distribution across the adult age range. Eligibility criteria included residing in the region for at least 6 months and the ability to provide informed consent. The GOS first recruited the female cohort between 1993 and 1997, with follow‐up assessments occurring at 2, 4, 6, 8, 10 and 15 years postbaseline. Recruitment for men occurred approximately a decade later, between 2001 and 2006, with follow‐up assessments conducted at 5 and 15 years. For this analysis, data were drawn from the baseline follow‐up for men (2001–2006) and the 6‐year follow‐up for women (2001–2003), which served as the baseline for analyses. Women were followed until 31 December 2022, providing approximately 19–21 years of follow‐up, while men were followed to the same date, providing approximately 16–20 years of follow‐up. The research obtained approval from the Human Research Ethics Committee at Barwon Health (IDs: 92/01 and 00/56), and all participants provided written, informed consent before their involvement.

### 2.2 Study Variables

#### 2.2.1 Outcome Variable

The outcome variable was the time to first emergency presentation due to a fall. These data were obtained via data linkage with the Victorian Emergency Minimum Dataset (VEMD). The VEMD contains data from major Victorian public hospitals with Emergency Departments, including the Warrnambool and District Base Hospital, Hamilton Base Hospital and University Hospital Geelong [28]. The linked data included emergency presentations from 1 January 2000 to 31 December 2022. Participants were followed from their baseline visit (for men) or from the 6‐year follow‐up (for women) until they experienced an emergency presentation for a fall, death or until the study's completion (31/12/2022). Mortality data were obtained via linkage with the National Death Index, managed by the Australian Institute of Health and Welfare.

#### 2.2.2 Exposure Variable

Hip abductor and flexor muscle strength were assessed using a handheld dynamometer (Nicholas manual muscle tester, model 01160, Lafayette Instrument Company), and values were adjusted for leg lean mass measured by dual‐energy X‐ray absorptiometry (DXA). A break test was used to assess hip muscle forces by applying force until the muscle could no longer resist, providing precise quantification of muscle resistance and the force required to initiate an isometric contraction. Hip flexion (HF) and abduction force were measured three times on each side without recovery periods to maintain consistency and accuracy. This approach is detailed further in the study by Pasco et al. [29]. The maximal HF and abduction forces were converted to Newtons (N) by multiplying by 9.81. The handheld dynamometer demonstrated excellent intrarater reliability for both hip abduction (HA) and HF strength. For HA, the intraclass correlation coefficient (ICC) was 0.936 (95% CI: 0.930–0.941) for a single trial and 0.978 (95% CI: 0.975–0.979) for the average of three trials, while for HF, the ICC was 0.898 (95% CI: 0.888–0.908) for a single trial and 0.964 (95% CI: 0.960–0.967) for the average of three trials, indicating that repeated measurements were highly reliable.

Appendicular lean mass (ALM) was determined using whole‐body DXA (Lunar Prodigy‐Pro, Madison, WI, USA), a method that provides detailed body composition measurements for the arms and legs. The DXA‐derived lean soft tissue mass is highly correlated with muscle mass measured by magnetic resonance imaging [30], as it encompasses tissues that are neither fat nor bone. This procedure is explained elsewhere by Pasco et al. [29]. For this analysis, the sum of lean mass from the right and left legs was used as the denominator to standardize muscle strength. Muscle‐specific strength (N/kg) was obtained by dividing muscle force (N) by leg lean mass (kg) [31].

#### 2.2.3 Covariates

Participants' weight and height were recorded with a precision of ±0.1 kg and ±0.1 cm, respectively, using electronic scales and a wall‐mounted Harpenden stadiometer. Body mass index (BMI) was subsequently computed as weight in kilogrammes divided by the square of height in metres (kg/m^2^). Smoking status and alcohol intake were self‐reported. Smoking was defined as the regular use of manufactured cigarettes, hand‐rolled cigarettes, cigars or a pipe. High alcohol intake was defined as > 2 standard drinks per day for women and > 3 for men, with one standard drink equal to 10 g [32].

Balance confidence was assessed using the 14‐item Modified Falls Efficacy Scale (MFES) [33], which measures confidence in performing daily activities without falling. Scores range from 0 to 140, with lower scores indicating lower balance confidence. The MFES is a highly reliable (Cronbach's alpha = 0.95; ICC = 0.93) and validated tool [33]. The Timed Up and Go (TUG) test assesses mobility and balance, with times ≥ 12 s indicating slow performance and < 12 s considered normal [34]. Mobility was self‐reported using a 7‐point scale as previously described [27], with categories of *very active*, *active*, *sedentary*, *limited*, *inactive*, *chair/bedridden* and *bedfast*. For analysis, mobility was grouped as high (*very active* and *active*) or low (*sedentary*, *limited*, *inactive*, *chair/bedridden* and *bedfast*). Participants' use of mobility assistive devices (e.g., walkers or canes) was recorded (yes/no) [32]. A fall was defined as an event in which an individual comes to rest on the ground unintentionally, from a lying, sitting or standing position [27]. Self‐reported previous falls within the previous 12 months were collected at baseline and classified as a binary variable (yes = experienced ≥ 1 fall; no = no falls). Polypharmacy was defined as the use of five or more medications daily [35].

### 2.3 Statistical Analysis

Participant characteristics and covariates were analysed using descriptive statistics. Continuous variables were tested for normality; normally distributed variables were reported as means with standard deviations (SD), while non‐normally distributed variables were summarized as medians with interquartile ranges (IQR). Categorical variables were presented as frequencies and percentages. Group differences were evaluated using two‐sample *t*‐tests or Mann–Whitney *U* tests for continuous variables, based on their distribution, and chi‐squared tests for categorical variables.

Pearson correlation coefficients were used to examine the linear relationships between HMS and selected continuous variables, including age, height, weight, MFES score and TUG test performance. Correlation coefficients are categorized as negligible (0.00–0.10), weak (0.10–0.39), moderate (0.40–0.69), strong (0.70–0.89) and very strong (0.90–1.00) [36].

In time‐to‐event analysis, such as evaluating the time to the first emergency presentation for an incident injurious fall, the standard Cox proportional hazards model is commonly used. However, our dataset contains a substantial number of deaths, which act as competing risks—events that preclude the occurrence of the primary outcome, in this case, fall‐related emergency presentation. As the proportion of deaths in our sample exceeds 10%, it is essential to account for them to avoid biased estimation of the risk of the primary outcome [37]. To address this, we employed Fine and Gray's competing risk regression model, which allows for appropriate analysis of the event of interest while accounting for the influence of competing events such as death.

Following model fitting, the proportional sub‐distribution hazard assumption was evaluated using multiple approaches, including interactions between covariates and time, as well as the Schoenfeld residual test. No violations of the assumption were detected. Potential covariates were identified based on theoretical reasoning informed by previous research. A bivariable competing risk regression identified fall‐related factors, with variables (*p* < 0.25) included in the multivariable model. The multivariable model was adjusted for demographic factors (age and sex), behavioural factors (smoking and alcohol intake), health conditions (polypharmacy and previous self‐reported falls), mobility‐related factors (mobility, use of mobility assistive devices and TUG test) and balance confidence (operationalized using MFES score). Both crude and adjusted sub‐distribution hazard ratios, along with their 95% confidence intervals (CI), were reported to assess associations. In the multivariable analysis, statistical significance was defined as a *p*‐value of less than 0.05.

A mediation model using structural equation modelling and 5000 bootstrap samples confirmed that hip abductor strength (HAS) mediates the link between balance confidence and incident injurious falls. A mediation analysis was performed with balance confidence as the exposure variable (X), HAS as the mediator (M) and incident injurious falls as an outcome variable (Y). The mediation effect was evaluated using the bootstrapping method with 5000 resampling iterations. The direct effect (DE) measures the effect of balance confidence without mediation or interaction, while the indirect effect (IE) reflects the effect solely due to mediation. The total effect is the sum of DE and IE [38]. The proportion mediated was calculated using the formula [(indirect effect) / (total effect) * 100] [38]. The data were analysed using Stata version 18 (StataCorp. 2023. Stata Statistical Software: Release 18. College Station, TX: StataCorp LLC).

## 3 Results

### 3.1 Participant Characteristics by Incident Injurious Fall Status

Among the variables examined, age, height and body mass were significantly different between fallers and nonfallers. Fallers were older than nonfallers, with a median age of 79 years (IQR: 71–84) compared to 76 years (IQR: 71–82; *p* < 0.001). They also had a slightly shorter average height (1.6 ± 0.1 m vs. 1.7 ± 0.1 m; *p* = 0.022) and lower average body mass (76.8 ± 14.7 kg vs. 79.0 ± 13.8 kg; *p* = 0.049) (Table 1).

**TABLE 1:** Characteristics of the participants. Data presented as median (IQR), mean (±SD) or *n* (%).

Variables	Injurious fall status		*p*
	No	Yes	
Age, yr	76 (70–82)	78 (73–84)	< 0.001
Height, m	1.68 ± 0.1	1.66 ± 0.1	0.015
Body mass, kg	79.0 ± 13.8	76.8 ± 14.7	0.049
Body mass index, kg/m^2^	27.9 ± 3.3	27.6 ± 3.6	0.243
Leg lean mass, kg	14.8 ± 4.6	14.7 ± 4.4	0.716
Relative leg lean mass, kg/m^2^	2.75 ± 0.6	2.71 ± 0.6	0.356
Timed Up and Go test, s	9.17 ± 3.0	9.55 ± 2.6	0.109
Low mobility	197 (27.1)	64 (29.2)	0.520
Use of a mobility assistive device	60 (8.3)	20 (9.2)	0.655
Smoking	57 (9.9)	19 (10.2)	0.905
High alcohol intake	269 (38.9)	69 (32.2)	0.077

Abbreviations: IQR = interquartile range; SD = standard deviation.

### 3.2 HMS by Fall Status and Sex

Participants who experienced injurious falls had lower HAS than nonfallers in both men (*p* = 0.012) and women (*p* = 0.008). No significant differences were observed in hip flexor strength (HFS) between fallers and nonfallers among men (*p* = 0.267) or women (*p* = 0.117) (Table 2).

**TABLE 2:** HMS (N/kg) by injurious fall status and sex, analysed using independent *t*‐tests. Data are presented as mean ± SD.

Variables	Men			Women		
	Nonfallers	Fallers	*p*	Nonfallers	Fallers	*p*
HMS						
Hip flexor	12.5 ± 4.9	11.0 ± 2.9	0.158	13.6 ± 3.9	12.7 ± 4.9	0.108
Hip abductor	8.1 ± 2.9	6.5 ± 1.9	**0.012**	5.9 ± 2.0	5.2 ± 1.7	**0.008**

Abbreviations: HMS = hip muscle strength; N/kg = Newtons/kilogramme; SD = standard deviation.

### 3.3 Correlation Analysis of HMS With Key Variables

HFS showed a weak negative correlation with TUG time (*r* = −0.212, *p* < 0.001). Weak positive correlations were observed between HFS and MFES score (*r* = 0.197, *p* < 0.001). In addition, HFS demonstrated a moderate positive correlation with HAS (*r* = 0.508, *p* < 0.001) (Table S1).

HAS showed weak negative correlations with age (*r* = −0.222, *p* < 0.001) and TUG time (*r* = −0.278, *p* < 0.001). HAS was weakly positively correlated with MFES score (*r* = 0.167, *p* < 0.005) and body weight (*r* = 0.276, *p* < 0.001), while a moderate positive correlation was observed with height (*r* = 0.352, *p* < 0.001) (Table S1).

### 3.4 Incidence of Injurious Falls

The median follow‐up duration was 11.5 years (IQR: 5.9–19.0), yielding a total of 11 369 person‐years of observation. Among men, the median follow‐up time was 9.3 years (IQR: 5.0–17.1), contributing 6085 person‐years, whereas women had a longer median follow‐up of 18.1 years (IQR: 8.7–20.3), contributing 5284 person‐years. During follow‐up, 125 men (13.1%) died, while 94 women (9.9%) experienced at least one injurious fall. The incidence rate of injurious falls was 20.5 per 1000 person‐years in men (95% CI: 17.2–24.5), 17.8 per 1000 person‐years in women (95% CI: 14.5–21.8) and 19.3 per 1000 person‐years overall (95% CI: 16.9–22.0).

### 3.5 HAS as a Predictor of Incident Injurious Falls

In unadjusted analyses, greater HAS was associated with a reduced risk of incident injurious falls (cSHR = 0.830, 95% CI: 0.749–0.919; *p* < 0.001). This association remained significant after adjustment for covariates (aSHR = 0.835, 95% CI: 0.724–0.963; *p* = 0.013). Each 1‐N/kg increase in HAS was associated with a 16.5% reduction in the sub‐distribution hazard of incident injurious falls. In contrast, HFS was not associated with incident injurious falls in either unadjusted (cSHR = 0.948, 95% CI: 0.884–1.018; *p* = 0.140) or adjusted models (aSHR = 1.037, 95% CI: 0.952–1.130; *p* = 0.407) (Table 3). Mediation analysis indicated that HAS accounted for 23.7% of the association between MFES score and the incidence of injurious falls.

**TABLE 3:** A competing risk regression analysis of hip muscle strength as a predictor of injurious falls.

Variables	Risk of injurious falls			
	cSHR (95% CI)	*p*	aSHR (95% CI)	*p*
Hip flexion strength	0.948 (0.884, 1.018)	0.140	1.037 (0.952, 1.130)	0.407
Hip abductor strength	0.830 (0.749, 0.919)	**< 0.001**	0.835 (0.724, 0.963)	**0.013**

*Note:* The multivariable model was adjusted for age, sex, alcohol use, smoking, polypharmacy, previous self‐reported falls, mobility, use of mobility assistive devices, Timed Up and Go test and balance confidence.

Abbreviations: aSHR = adjusted sub‐distribution hazard ratio; CI = confidence interval; cSHR = crude sub‐distribution hazard ratio.

## 4 Discussion

In this prospective study, 13.1% of men and 9.9% of women experienced at least one injurious fall, and greater HAS was independently associated with a lower risk of incident injurious falls, whereas HFS was not. Each 1‐N/kg increase in HAS was associated with a 16.5% reduction in the sub‐distribution hazard of injurious falls, while HFS showed no significant association. HAS mediated 23.7% of the association between MFES score and incident injurious falls, suggesting that muscle deconditioning associated with lower balance confidence contributes to injurious falls. Together, these findings identify HAS as a key modifiable target for injurious fall prevention.

The findings of de Almeida Nagata et al. [23] align with our results, underscoring the strong protective effect of HAS against falls. Their study reported an 86.3% reduction in fall risk per 1‐Nm/kg increase, while we observed a 16.5% reduction per 1‐N/kg increase, supporting the robustness of this association. Despite this consistency, notable discrepancies in effect size were evident, likely attributable to differences in age criteria, fall ascertainment methods and follow‐up durations. First, the study populations varied in age criteria, with our study including participants aged ≥ 65 years, while de Almeida Nagata et al. [23] included individuals aged ≥ 60 years. Second, the methods used to ascertain falls differed considerably; our study employed objective data linkage to capture fall‐related events, whereas de Almeida Nagata et al. [23] relied on self‐reported falls collected through monthly telephone interviews. Third, the duration of follow‐up was substantially longer in our study—up to 22 years—compared to just 1 year in the study by de Almeida Nagata et al. [23], potentially contributing to differences in incidence rates and observed associations.

According to the Systems Theory of Postural Control, the protective role of HAS arises from its contribution to the coordinated integration of sensory, motor and musculoskeletal components required for balance [39]. Within this framework, hip abductors are crucial for lateral stability, and their weakness can cause poor control of body balance, increasing the risk of side or backward falls—commonly linked to hip fractures and serious injuries [40]. The gluteus medius and minimus play a key role in maintaining pelvic alignment during gait. Weakness in these muscles can result in compensatory trunk movements and altered gait, which may increase instability while walking [41]. The continued link between HAS and falls after adjustment indicates that this muscle group may be a key independent predictor of falls [23].

Hip flexors, like the iliopsoas, are mainly involved in initiating movement by lifting the thigh during walking or rising from a chair, but they play a limited role in maintaining balance during sudden postural disturbances [42]. While weakness may reflect reduced activity [43] or sarcopenia, it does not directly increase fall events as hip abductors do, as these muscles play a lesser role in stabilizing the body during lateral or backward movements, where most falls tend to occur.

Weakness in the hip abductors is more strongly linked to fall risk than hip flexor weakness, as falls often stem from loss of lateral or backward balance [40]. This is likely because the hip abductors are essential for maintaining stability during side‐to‐side and backward movements, particularly when responding to unexpected shifts in balance. Recognizing the distinct roles of hip muscles in movement and balance provides important guidance for fall prevention. While strengthening the hip flexors can enhance mobility, focusing on the hip abductors may be even more critical for reducing fall risk, especially among older adults. Exercise targeting lower limb muscles is a beneficial intervention for fall prevention [44], as lower extremity strengthening and balance exercises significantly improve strength, balance and mobility, leading to reduced fall rates, injurious falls and emergency department visits [45].

Our findings indicate that lower balance confidence was linked to reduced hip strength, which mediated about 23.7% of its effect on injurious falls—indicating that part of the psychological impact operates through diminished physical function. Balance confidence often leads older adults to limit their mobility and avoid physical activity [26]. This reduction in movement can result in muscle deconditioning, particularly in the lower limb muscles such as the hip abductors, which are essential for balance and stability [46]. Prolonged disuse of the hip abductor muscles leads to weakness, especially impacting balance and lateral stability during walking [47]. These muscles play a vital role in stabilizing the pelvis during single‐leg stance—a critical phase of gait [48]—so their weakness can increase the risk of falling.

Our findings advance existing research by establishing the mediating role of HAS in the association between balance confidence and injurious fall, using a robust competing risk regression model combined with bootstrapped mediation analysis. This approach offers a more complex understanding of the interplay between psychological and physical determinants of falls. The results underscore the potential effectiveness of multifaceted interventions that concurrently target balance confidence and muscular strength deficits, particularly in the hip abductors. These findings highlight the value of routinely assessing HAS in geriatric care to identify older adults at increased risk of falls. Simple methods, such as side‐lying leg lifts or handheld dynamometry, can be used for screening and fall prevention programmes, which should incorporate hip‐targeted resistance training to reduce fall risk and enhance physical function [49].

### 4.1 Strengths and Limitations

Fall events were objectively identified through linkage with the VEMD, reducing recall bias. Participants were drawn from the well‐established GOS, providing standardized longitudinal data. Hip muscle force and leg lean mass were measured objectively using handheld dynamometry and DXA, respectively. Balance confidence was assessed using the validated MFES tool, and the analysis accounted for the cohort's high mortality through competing risk regression. Bootstrapped mediation analysis provided novel insights into how HAS mediates the relationship between balance confidence and the incidence of injurious falls.

The relatively small sample size may have reduced statistical power, and findings should be interpreted with caution. The cohort was drawn from specific regions in southeastern Australia, which may limit generalizability due to regional, cultural and lifestyle differences. Muscle force and lean mass were measured only once, preventing assessment of changes over time. Falls were captured only if they led to emergency presentations, likely underestimating total fall incidence by missing minor events. Although mobility was assessed, the absence of objective physical activity data may have confounded the association between HAS and falls; future studies should incorporate validated measures such as the Physical Activity Scale for the Elderly (PASE). Finally, the cross‐sectional assessment of MFES scores and HAS limits causal inference, including mediation analysis. Larger longitudinal studies with repeated measurements of balance confidence and muscle strength are needed to clarify these pathways.

## 5 Conclusion

Greater HAS reduced the sub‐distribution hazard of injurious falls and partially mediated the relationship between balance confidence and injurious falls. These findings highlight the importance of targeted hip abductor strengthening in fall prevention, alongside interventions that address psychological factors related to balance confidence. Integrating physical strengthening with strategies to enhance balance confidence may provide the most effective approach to reducing injurious falls among older adults.

## Funding

T.Y. was supported by a Deakin University Postgraduate Research Scholarship (DUPRS). J.A.P. has received funding from the National Health and Medical Research Council (NHMRC). K.L.H‐K. was supported by an Alfred Deakin Postdoctoral Research Fellowship.

## Consent

The authors have nothing to report.

## Conflicts of Interest

The authors declare no conflicts of interest.

## Supporting information


**Table S1:** Correlation matrix for age, anthropometry, mobility, MFES score and hip flexion and abductor strength.

## Data Availability

The data that support the findings of this study are available from the Victorian Minimum Emergency Dataset and the Geelong Osteoporosis Study. Restrictions apply to the availability of these data, which were used under approvals for this study.
