# Supplementary material for: Hip Abductor Strength Predicts Injurious Falls and Mediates the Balance Confidence–Falls Relationship: A Competing Risk Model
Source: J Cachexia Sarcopenia Muscle. 2026 Apr 1;17(2):e70280. doi: 10.1002/jcsm.70280 (PMC13045402; doi:10.1002/jcsm.70280)
Supplement: Supplementary file 1 — Table S1: Correlation matrix for age, anthropometry, mobility, MFES score and hip flexion and abductor strength. [file JCSM-17-e70280-s001.docx]

Supplementary Table 1: Correlation matrix for age, anthropometry, mobility, MFES score, and hip flexion and abductor strength.

| Variables | Age | Height | Weight | TUG test | MFES score | HFS | HAS |
| --- | --- | --- | --- | --- | --- | --- | --- |
| Age | 1 |  |  |  |  |  |  |
| Height | -0.208^**^ | 1 |  |  |  |  |  |
| Weight | -0.321^**^ | 0.790^**^ | 1 |  |  |  |  |
| TUG test | 0.267^**^ | -0.004 | -0.049 | 1 |  |  |  |
| MFES score | -0.169^**^ | 0.023 | 0.055 | -0.438^**^ | 1 |  |  |
| HFS | -0.010 | -0.049 | -0.065 | -0.212^**^ | 0.197^**^ | 1 |  |
| HAS | -0.222^**^ | 0.352^**^ | 0.276^**^ | -0.278^**^ | 0.167^*^ | 0.508^**^ | 1 |

TUG test: Timed Up and Go test; HFS: Hip flexion strength; HAS: Hip abduction strength; ^**^P<0.001; ^*^P<0.005
